# Augmenting Deep Learning-Based PSMA PET/CT Metastasis Segmentation With a Population-Level Spatial Atlas

**DOI:** 10.64898/2026.08.26.26361439

**Published:** 2026-08-31

**Authors:** Grant Chau, Bashirul Azam Biswas, Biratal Raj Wagle, Matthew E. Maeder, James B. Yu, Indrani Bhattacharya

## Abstract

Automated lesion segmentation is increasingly central to PSMA PET/CT interpretation, supporting staging, treatment planning, and response assessment at a scale that outpaces available nuclear-medicine expertise. However, automated PSMA-PET/CT whole-body lesion segmentation models are trained on images alone, with no knowledge of where in the body prostate metastases actually tend to occur. Radiologists use clinical domain knowledge of metastatic spread, but its absence in machine learning models produces false positives in anatomically implausible locations and missed lesions in high-risk sites such as the liver. In this work, we explore whether population-level spatial knowledge of metastatic spread can be used to augment deep learning segmentation predictions, and how such a prior should be fused with a network’s output, without additional training. We build a data-driven metastasis atlas from 375 expert-annotated whole-body PSMA PET/CT scans and investigate its fusion with a trained segmentation network under a Bayesian framework, in which prediction probabilities from an nnU-Net-based lesion segmentation model serve as the likelihood and the data-driven atlas as the prior. Because metastases occupy only a small fraction of whole-body voxels, the atlas’s peak probability is too low, and standard power-scaled or naive Bayesian pooling references lack the tools to deal with this shortcoming. This causes these standard fusion strategies to fail and, in the naive Bayesian case, to sharply degrade performance. We instead derive a calibrated, background-referenced log-odds fusion, one of many possible approaches to combine a population atlas with a deep learning model’s predictions, distinct from classical multi-atlas label fusion in that it fuses a single population prior with a trained network’s softmax rather than combining several registered atlases. Furthermore, this approach is neutral outside atlas support by construction, reduces exactly to the baseline network when unweighted, and requires no retraining. This atlas fusion significantly improved mean Dice over the baseline nnU-Net on a disjoint internal test set (+0.011, Holm-adjusted *p* = 0.021) and on an independent external cohort (+0.0129, Holm-adjusted *p* = 3.8×10 ^*−* 16^ ), with lesion sensitivity improving from 0.849 to 0.861 internally and Dice improving over baseline in every stratified anatomic region, including the rare, high-risk sites motivating this work, while naive Bayesian pooling degrades performance sharply and power-scaled pooling underperforms it throughout. Our findings suggest that population-level spatial priors can meaningfully augment deep learning predictions in whole-body oncologic segmentation, provided the fusion rule is calibrated to where the prior actually carries signal.

## Full Text Availability

The license terms selected by the author(s) for this preprint version do not permit archiving in PMC. The full text is available from the preprint server.

